# Supplementary material for: Effects of Serotonin on Cell Viability, Permeability of Bovine Mammary Gland Epithelial Cells and Their Transcriptome Analysis
Source: Int J Mol Sci. 2023 Jul 13;24(14):11388. doi: 10.3390/ijms241411388 (PMC10379418; doi:10.3390/ijms241411388)
Supplement: Supplementary file 1 [file ijms-24-11388-s001.zip › ijms-2468135-supplementary.pdf]

Table S1. primer sequences of selected genes.

Gene Name	Gene Symbol	GenBank Accession No.	Primer Sequence (5′-3′)
Claudin 1	CLDN-1	NM_001001854.2	F:CGGTCAATGCCAGGTATG<br>R:CTGGGTGTTGGGTAAGATG
Claudin 4	CLDN-4	NM_001014391.2	F:CCAACTGTGTGGATGATGAG<br>R:CGCGGATGACGTTGTTAG
Claudin 8	CLDN-8	NM_001098096.1	F:GAGAGTGTCTGCCTTCATTG<br>R:AGCAGCGAGTCGTAGATT
ZO-1	TJP1	XM_015468497.1	F:CTTTACGAGCTCCAGGCACT<br>R:GGGGTCCTTCCTGTACACCT
ZO-2	TJP2	NM_001102482.1	F:GGACTCGGACAAGCCTAT<br>R:GACCGGTTCTGGTCATTTC
E-cadherin	CDH1	NM_001002763.1	F:GCCAACGAGCTGATACAC<br>R:CGGCATGAGAGAAGAGAATG
Transforming growth factor beta 1	TGF-β1	NM_001166068.1	F:GGACCTGGGCTGGAAGTG<br>R:CTGCTCCACCTTGGGCTT
Caspase-3	CASP3	NM_001077840.1	F:AAGCCATGGTGAAGAAGGAA<br>R:CCTCAGCACCACTGTCTGTC
Stearoyl-CoA desaturase	SCD-1	NM_173959.4	F: CTACACAACCACCACCACCA<br>R: CAGGGCACCCATCAGATAGT
Fatty acid synthase	FASN	NM_001012669.1	F:CCCTGAATGTGAGGCAGTGTG<br>R:TTAGCTGTGGTGAGGAGCCA
Acetyl-CoA carboxylase alpha	ACACA	NM_174224.2	F:CTTCTGTGATTCCCCACCCC<br>R:GTTCATCCCTGGGGACCTTG
Glyceraldehyde-3-phosphate dehydrogenase	GAPDH	NM_001034034.2	F:GATGCTGGTGCTGAGTATG<br>R:CAGAAGGTGCAGAGATGATG
Ribosomal protein S9	RPS-9	NM_001101152.2	F:GGAGACCCTTCGAGAAGTCC<br>R:CTTTCTCATCCAGCGTCAGC
